# Supplementary material for: Sorting at embryonic boundaries requires high heterotypic interfacial tension
Source: Nat Commun. 2017 Jul 31;8:157. doi: 10.1038/s41467-017-00146-x (PMC5537356; doi:10.1038/s41467-017-00146-x)
Supplement: Supplementary file 2 — Supplementary Software 1 [file 41467_2017_146_MOESM2_ESM.zip › PottsModel/SrcPottsModel/doc/model/Constants.LatticeManager.html]

Constants.LatticeManager


---


|  |  |  |
| --- | --- | --- |
| **Overview** \| **Package** \| **Class** \| **Use** \| **Tree** \| **Deprecated** \| **Index** \| **Help** | |  |
| **PREV CLASS**   **NEXT CLASS** | **FRAMES**    **NO FRAMES**     **All Classes** |  |
| SUMMARY: NESTED \| FIELD \| CONSTR \| METHOD | DETAIL: FIELD \| CONSTR \| METHOD |  |


---


## model Class Constants.LatticeManager

```
java.lang.Object
  model.Constants.Manager
      model.Constants.LatticeManager
```

**Enclosing class:**: Constants

---

``` private class Constants.LatticeManager extends Constants.Manager ```

---

| **Field Summary** | |
| --- | --- |
| `private  int` | `cellSize` |
| `private  boolean` | `latticeBuilt` |
| `private  int` | `latticeHeight` |
| `private  Lattice.Type` | `latticeType` |
| `private  int` | `latticeWidth` |
| `private  boolean` | `makeRound` |
| `private  int` | `mediumSize` |

**Fields inherited from class model.Constants.Manager**

`prop`


**Constructor Summary**

`Constants.LatticeManager(java.util.Properties p)`


| **Method Summary** | |
| --- | --- |
| `(package private)  void` | `processProperties()` |

**Methods inherited from class java.lang.Object**

`clone, equals, finalize, getClass, hashCode, notify, notifyAll, toString, wait, wait, wait`

**Field Detail**

### latticeBuilt

```
private boolean latticeBuilt
```

---


### latticeType

```
private Lattice.Type latticeType
```

---


### latticeWidth

```
private int latticeWidth
```

---


### latticeHeight

```
private int latticeHeight
```

---


### cellSize

```
private int cellSize
```

---


### mediumSize

```
private int mediumSize
```

---


### makeRound

```
private boolean makeRound
```


**Constructor Detail**

### Constants.LatticeManager

```
public Constants.LatticeManager(java.util.Properties p)
```


**Method Detail**

### processProperties

```
void processProperties()
```

:   **Specified by:**: `processProperties` in class `Constants.Manager`


---


|  |  |  |
| --- | --- | --- |
| **Overview** \| **Package** \| **Class** \| **Use** \| **Tree** \| **Deprecated** \| **Index** \| **Help** | |  |
| **PREV CLASS**   **NEXT CLASS** | **FRAMES**    **NO FRAMES**     **All Classes** |  |
| SUMMARY: NESTED \| FIELD \| CONSTR \| METHOD | DETAIL: FIELD \| CONSTR \| METHOD |  |


---
